# Risk of COVID-19 Infection After Full Immunization in Patients With Inflammatory Bowel Disease on Treatment: A Research Network Analysis

**DOI:** 10.7759/cureus.34004

**Published:** 2023-01-20

**Authors:** Julton Tomanguillo, Lauren Searls, Frank H Annie, Suzanne Kemper, Vishnu Naravadi

**Affiliations:** 1 Internal Medicine, Charleston Area Medical Center (CAMC), Charleston, USA; 2 Cardiology, Charleston Area Medical Center (CAMC), Charleston, USA; 3 Outcomes Research, Charleston Area Medical Center (CAMC) Health Education and Research Institute, Charleston, USA; 4 Gastroenterology, Charleston Area Medical Center (CAMC), Charleston, USA

**Keywords:** biologic/small molecule, us food and drug administration, sars-cov2, steroids, ibd

## Abstract

Background: Acute respiratory syndrome coronavirus-2 (SARS-CoV-2) is an issue in treating patients with Inflammatory Bowel Disease (IBD) due to concerns for infection risk and poor post-vaccination antibody response. We examined the potential impact of IBD treatments on SARS-CoV-2 infection rates after full immunization against COVID-19.

Methods: Patients who received vaccines between January 2020 and July 2021 were identified. The post-immunization Covid-19 infection rate at 3 and 6 months was assessed in IBD patients receiving treatment. The infection rates were compared to patients without IBD.

Results: The total number of IBD patients was 143,248; of those (n=9405), 6.6% were fully vaccinated. In IBD patients taking biologic agents/small molecules, no difference in Covid-19 infection rate was found at 3 (1.3% vs. 0.97%, p=0.30) and 6 months (2.2% vs. 1.7%, p=0.19) when compared to non-IBD patients. No significant difference in Covid-19 infection rate was found among patients receiving systemic steroids at 3 (1.6% vs. 1.6%, p=1) and 6 months (2.6% vs. 2.9%, p=0.50) between the IBD and non-IBD cohorts.

Conclusions: The COVID-19 immunization rate is suboptimal among IBD patients (6.6%). Vaccination in this cohort is under-utilized and should be encouraged by all healthcare providers.

## Introduction

Multiple medical societies have published vaccine guideline recommendations for patients with Inflammatory Bowel Disease (IBD) [1-4]; however, despite these guidelines, the vaccination rate among IBD patients has been meager. It is estimated that among IBD patients, only 43.5% received influenza immunization, 24.1% pneumococcal vaccination, 48.3% Hepatitis B vaccination, 43.8% Measles, Mumps, Rubella (MMR) vaccine, 43% DTaP, 2.1% herpes zoster, and 3.4% Human papillomavirus (HPV) vaccination [1-2]. The current COVID-19 pandemic is a significant challenge for patients with IBD. Recent studies have shown that the outcomes of patients with IBD and COVID-19 infection compared to Covid-19 infection in the general population are similar [3-4]. Three vaccines have received full approval from the US Food and Drug Administration in the US. These are efficacious and safe in preventing Covid-19 infection [5-9]. Hadi et al. identified 3,866 IBD patients who received immunization against COVID-19; of these, 113 patients reported adverse events (2.03%), which is a similar and comparable rate among patients without IBD (relative risk [RR], 2.50; 95% Q6 confidence interval [CI], 2.07-3.00) [8]. Additionally, medical societies such as the British Society of Gastroenterology IBD Section and IBD Clinical Research Group strongly recommended the Covid-19 vaccine for patients with IBD [9]. This study examines the COVID-19 immunization rate and the potential impact of IBD treatments on Covid-19 infection rates after receiving full immunization.

This article was previously presented as a meeting abstract at the 2022 AASLD Annual Scientific Meeting From Nov 4-8, 2022. 

## Materials and methods

We identified patients aged 18 to 90 who received the BNT162b2 (Pfizer-BioNTech), mRNA-1273 (Moderna), and Ad26.COV2.S (Johnson & Johnson) cases between January 20, 2020, and July 1, 2021. The post-immunization Covid-19 infection rate was compared at 3 and 6 months between non-IBD versus IBD patients receiving treatment with biologic/small molecule, immunomodulators, systemic steroids, or other agents. Propensity-matched (PSM) was used to examine the cohorts to control for potential confounders. We queried TriNetx (Covid-19 research network), a collection of 66 healthcare organizations from 6 countries. The Trinetx Inc. (Cambridge, MA) database is a global federal research network that combines real-time data from electronic medical records. The platform combines international data sets in a user-friendly interface that allows users to query different cohorts. A total of 143,248 IBD cases were identified. The Covid-19 infections database was queried; see a description of codes (supplement 1). Patients with an ICD-10 diagnosis of IBD, as listed in supplement 1, were identified. Additionally, IBD patients were grouped by the treatment they received. Treatment groups were as follows: 1. Biologic/small molecule (Certolizumab, Ustekinumab, Infliximab, Adalimumab, Vedolizuman, Tofacitinib, Golimumab), 2. Immunomodulators (Methotrexate, Azathioprine), and 3. Systemic steroids. The TriNetX platform uses several descriptive statistics as frequencies with percentages for differing categorical variables and mean ± standard deviation for continuous measures. The baseline characteristics were compared using Pearson's chi-squared test for defined categorical variables. To account for possible differences in the cohorts, a 1:1 PSM was created for well-matched groups, as shown (tables 1-3). The 1:1 PSM uses a logistic regression from the Python libraries (NumPy and Sklearn). This platform compares the results to R for verification. The final step in the verification process uses the nearest neighbor function set to a tolerance level of 0.01 and a deference value >0.1. Mortality was determined by the difference in association using the Kaplan-Meier method and the statistical difference between the risk factors.

**Table 1:** IBD on biologic/small molecule / No-IBD biologic/small molecule IBD-Inflammatory Bowel Disease, COPD-chronic obstructive pulmonary disease, CAD-coronary artery disease, CHF- Congestive heart failure, BMI-Body Mass Index

Baseline Characteristics	Unmatched Cohorts				Propensity Matched Cohorts			
	IBD on biologic/small molecule (N=2,443)	No-IBD biologic/small molecule (N=4,362)	P-Value	Standardized Mean Difference	IBD on biologic/small molecule (N=2,149)	No-IBD biologic/small molecule (N=2,149)	P-Value	Standardized Mean Difference
Age at Index	48±17.2	55.7± 14.7	<0.01	0.48	50.4±16.6	50.7±15.5	0.54	0.02
White	76.91%	70.59%	<0.01	0.14	75.24%	76.27%	0.43	0.03
Female	52.72%	67.03%	<0.01	0.30	57.28%	56.96%	0.83	0.01
Male	47.28%	32.94%	<0.01	0.30	42.72%	43.00%	0.85	0.01
Black or African American	11.01%	13.60%	0.02	0.08	11.45%	11.03%	0.66	0.01
Asian	3.03%	2.91%	0.78	0.01	3.16%	2.98%	0.72	0.01
Hypertension	35.86%	48.79%	<0.01	0.26	39.65%	39.23%	0.78	0.01
History of Smoking	21.70%	18.87%	0.01	0.07	21.50%	20.66%	0.50	0.02
Diabetes	13.22%	22.15%	<0.01	0.24	14.80%	13.26%	0.15	0.04
CAD	8.19%	11.44%	<0.01	0.11	9.12%	7.82%	0.13	0.05
COPD	5.36%	8.62%	<0.01	0.13	6.05%	5.17%	0.21	0.04
CHF	4.30%	6.69%	<0.01	0.11	4.79%	4.19%	0.34	0.03
Personal history of stroke	3.32%	3.92%	0.21	0.03	3.58%	3.21%	0.50	0.02
BMI > 30	26.24%	37.78%	<0.01	0.47	29.36%	28.80%	<0.01	0.02

**Table 2:** IBD on Immunomodulators / No-IBD Immunomodulators IBD-Inflammatory Bowel Disease, COPD-chronic obstructive pulmonary disease, CAD-coronary artery disease, CHF- Congestive heart failure, BMI-Body Mass Index

Baseline Characteristics	Unmatched Cohorts				Propensity Matched Cohorts			
	IBD on Immunomodulators (N=1,226)	No-IBD Immunomodulators (N=17,909)	P-Value	Standardized Mean Difference	IBD on Immunomodulators (N=1,224)	No-IBD Immunomodulators (N=1,224)	P-Value	Standardized Mean Difference
Age at Index	51±17.1	59.1± 14.8	<0.01	0.51	51±17.1	50.8±17.1	0.73	0.01
White	78.55%	62.91%	<0.01	0.35	78.51%	79.00%	0.77	0.01
Female	53.51%	59.41%	<0.01	0.12	53.60%	53.76%	0.94	0.03
Male	46.49%	40.58%	<0.01	0.12	46.41%	46.24%	0.94	0.01
Black or African American	11.42%	21.63%	<0.01	0.28	11.44%	12.01%	0.66	0.02
Asian	2.69%	3.55%	0.12	0.05	2.70%	2.04%	0.29	0.04
Hypertension	44.37%	68.34%	<0.01	0.50	44.44%	45.83%	0.49	0.03
History of Smoking	22.35%	23.53%	0.35	0.03	22.22%	19.85%	0.15	0.06
Diabetes	18.19%	36.91%	<0.01	0.43	18.22%	18.38%	0.92	0.01
CAD	12.15%	24.85%	<0.01	0.33	12.17%	12.34%	0.90	0.01
COPD	8.16%	19.02%	<0.01	0.32	8.17%	6.94%	0.25	0.05
CHF	6.20%	11.31%	<0.01	0.18	6.21%	5.39%	0.39	0.04
Personal history of stroke	3.83%	6.94%	<0.01	0.14	3.84%	3.02%	0.27	0.05
BMI > 30	32.22%	36.23%	<0.01	0.22	32.27%	32.27%	0.01	0.20

**Table 3:** IBD on Systemic Steroids / No-IBD Systemic Steroids IBD-Inflammatory Bowel Disease, COPD-chronic obstructive pulmonary disease, CAD-coronary artery disease, CHF- Congestive heart failure, BMI-Body Mass Index

Baseline Characteristic	Unmatched Cohorts				Propensity Matched Cohorts			
	IBD on Systemic Steroids (N=2,701)	No-IBD Systemic Steroids (N=100,370)	P-Value	Standardized Mean Difference	IBD on Systemic Steroids (N=2,701)	No-IBD Systemic Steroids (N=2,701)	P-Value	Standardized Mean Difference
Age at Index	55.6±17.8	59.8± 16.4	<0.001	0.242	55.6±17.8	55.8±17.7	0.77	0.01
White	78.82%	69.94%	<0.001	0.20	78.82%	79.67%	0.44	0.02
Female	58.65%	61.31%	0.01	0.10	58.65%	58.61%	0.98	0.01
Male	41.36%	38.68%	0.05	0.10	41.36%	41.39%	0.98	0.01
Black or African American	11.22%	17.55%	<0.001	0.18	11.22%	11.14%	0.93	0.02
Asian	2.70%	2.95%	0.45	0.02	2.70%	2.26%	0.29	0.03
Hypertension	53.31%	58.09%	<0.01	0.10	53.31%	53.39%	0.96	0.01
History of Smoking	28.36%	23.22%	<0.01	0.12	28.36%	28.25%	0.93	0.03
Diabetes	22.29%	25.41%	0.02	0.10	22.29%	22.40%	0.92	0.03
CAD	17.96%	19.56%	0.04	0.04	17.96%	17.25%	0.50	0.02
COPD	14.40%	18.96%	<0.01	0.12	14.40%	13.81%	0.53	0.02
CHF	12.07%	13.55%	0.03	0.04	12.07%	10.48%	0.10	0.10
Personal history of stroke	5.52%	5.68%	0.72	0.01	5.52%	4.26%	0.04	0.10
BMI > 30	32.54%	33.19%	<0.01	0.31	32.54%	32.32%	0.90	0.34

To understand if differing health conditions are affecting the outcomes of this study, a sensitivity analysis was performed to control for the possibility of residual confounders. The falsification endpoint of bleeding was used to see if there were possible confounders in each model.

## Results

**Figure 1:**
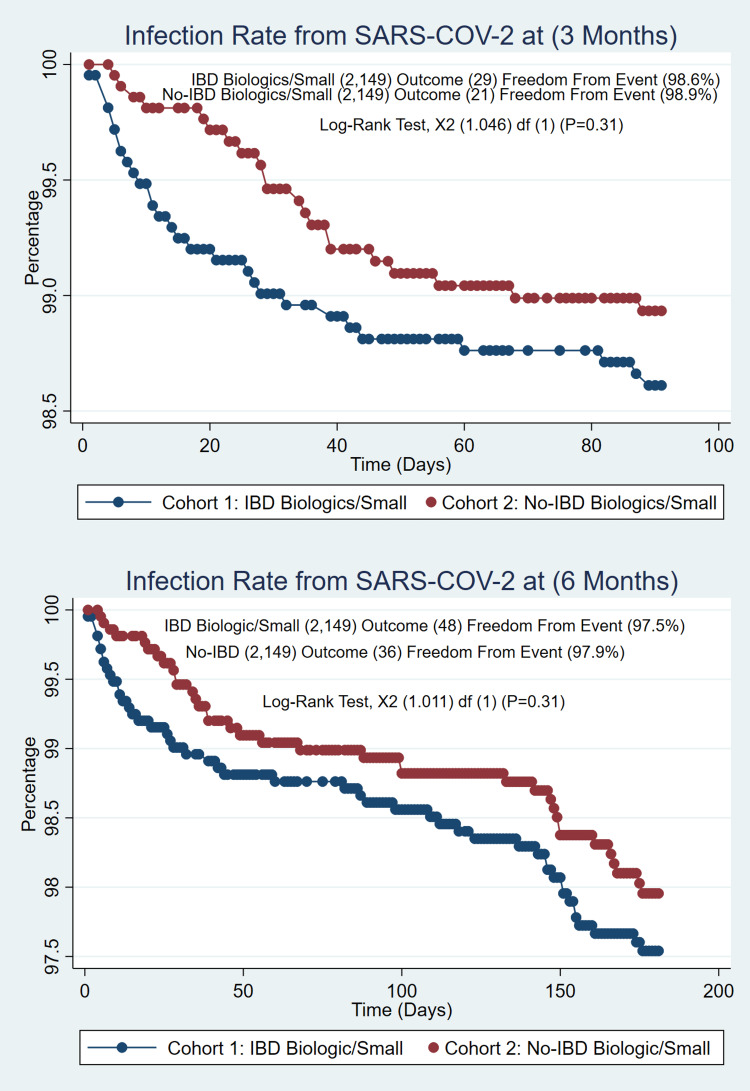
No-IBD Patients on Biologic/Small Molecule Treatments

**Figure 2:**
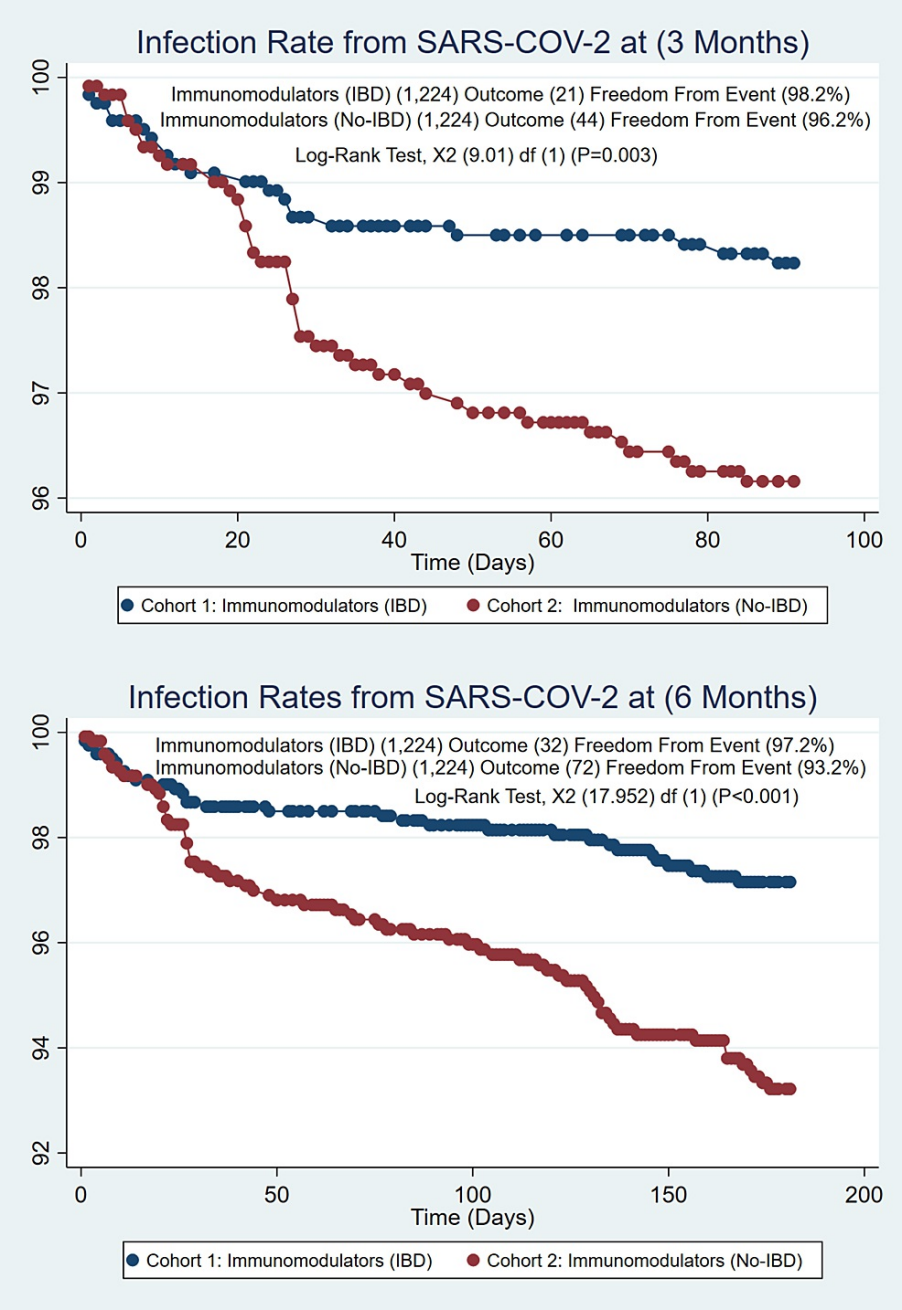
IBD Patients on Immunomodulators

**Figure 3:**
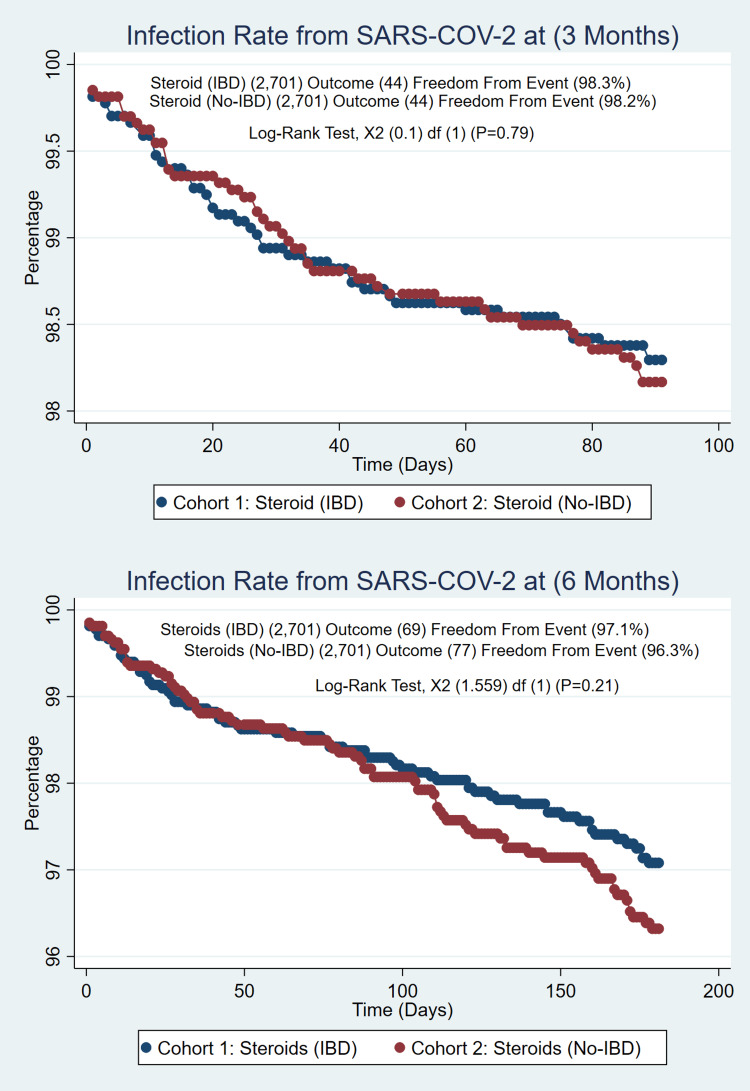
IBD Patients on Systemic Steroids

The total number of IBD cases identified was 143,248; of those, (n=9405) 6.6% were fully vaccinated (2 doses of Pfizer-BioNTech, 2 doses of Moderna, and 1 dose of Johnson & Johnson), and (n=873) 0.01% were partially vaccinated (single dose of Pfizer-BioNTech or Moderna). IBD cases on biologic/small molecule as treatment (n=2443) were compared with the non-IBD cases on the biologic/small molecule population (n=4632), which created a well-matched PSM of (n=2149/2149). No difference was found for the COVID-19 infection rate at 3 months (1.3% vs. 0.97%, p=0.30) and 6 months (2.2% vs. 1.7%, p=0.19) with similar incidences in IBD patients, as seen in Figure 1. Patients on immunomodulators (n=1224) were compared with the non-IBD population (n=17,909), which created a well-matched PSM of (1224/1224). The COVID-19 infection rate at 3 months (1.72% vs. 3.6%, p=0.003) and 6 months (2.6% vs. 5.9%, P=<0.001) showed a significant difference between these groups with higher rates of infection in non-IBD patients as seen in Figure 2. Patients on systemic steroids (n=2701) were compared with the non-IBD population (n=100,370); this created a well-matched PSM of (2701/2701). The Covid-19 infection rate at 3 months (1.6% vs. 1.6%, p=1.00) and 6 months (2.6% vs. 2.9%, p=0.50) were similar; no differences were found, as in figure 3.

The falsification endpoint of bleeding was used to see if there were possible confounders in each model. No difference in the falsification endpoint of any of the models was observed.

## Discussion

The rapid spread of the COVID-19 pandemic has significantly impacted all aspects of medicine. The pandemic has also challenged the management of IBD, as many patients require immunomodulatory therapies that might weaken the immune system, leading to an increased risk of infections. Multiple studies have been done to evaluate whether IBD patients have an increased risk of developing COVID-19 infection than the general population; there have been contradicting results [10-12]. Nevertheless, the primary focus should remain improving IBD patients' quality of life by achieving appropriate control of symptoms and encouraging medication adherence during difficult times. Patients with IBD should be vaccinated against COVID-19 at the earliest opportunity, as recommended by international advisory committees, and vaccination should not be delayed because a patient is receiving immune-modifying therapies [13]. The speed with which Covid-19 vaccines have been developed and the lack of data on long-term safety have been cited as reasons patients may have reduced vaccine acceptance, as demonstrated by a survey of Italian patients [14-15]. Historically, vaccines are safe and efficacious in IBD patients; however, the rate of immunization in this population is poor. Lack of patient education and the importance of vaccination being overlooked by general practitioners have contributed to the vaccination disparity in this population [15]. Only 6.6% of IBD patients in our study had full COVID-19 immunization, even though multiple authors have demonstrated that COVID-19 immunization is safe among these patients [8-9]. The role of primary care physicians and gastroenterologists in promoting prompt immunization is, therefore, of vital importance. The disease-related immune disorder and the immunosuppression induced by medications are two mechanisms that potentially compromise the natural response to immunization and impact the immunogenicity in IBD patients and, therefore, potentially increase the risk of viral infections [2,16-17]. Our study found that regardless of the treatment patients with IBD received, the rate of Covid-19 infection in IBD patients is similar to that of non-IBD patients; this trend has been expressed in the medical literature. A significant strength of our study is the use of an extensive database of 66 different healthcare facilities allowing us to include a large sample of 143,248 IBD patients and giving us a more representative sample with a diverse array of patients in the US and worldwide. Our study has some limitations due to the retrospective nature and the sizeable de-identified database nature of TriNetx; furthermore, patients who were not tested for Covid-19 and were asymptomatic could have been positive for COVID-19.

## Conclusions

In conclusion, the Covid-19 immunization rate among IBD patients was suboptimal (6.6%). Additionally, no increased risk of Covid-19 infection post-vaccination in IBD patients on immunosuppression was noted. This suggests durable protection for at least 6 months post-vaccination, likely from appropriate antibody response after vaccination. An essential task in reducing IBD patients' hesitancy to be vaccinated against Covid-19 not only falls in the hands of primary care physicians or gastroenterologists but is also the responsibility of any healthcare provider involved in the care of this population.

## Appendices

**Table 4:** List of Codes Used to Identify the Study Cohort Claim Evidence of COVID Infection

B34.2	Coronavirus infection unspecified
B97.29	Other coronavirus as the cause of diseases classified elsewhere
J12.81	Pneumonia due to SARS - associated coronavirus
U07.1	2019 -nCoV acute Respiratory disease (WHO) Laboratory Evidence of COVID Infection
94307-6	SARS coronavirus 2N gene (Nucleic acid amplification using primer- probe set N1)
94308-4	SARS coronavirus 2N gene (Nucleic acid amplification using primer- probe set N2)
94310-0	SARS - like Coronavirus N gene (Presence in Unspecified specimen by NAA with prove detection
94314-2	SARS coronavirus 2 RdRp gene (presence) in unspecified specimen by NAA with probe detection
94315-9	SARS coronavirus 2 E gene (presence) in unspecified specimen by NAA with probe detection
94316-7	SARS coronavirus 2N gene (presence in unspecified specimen by NAA with probe detection
J12.81	Pneumonia due to SARS - associated coronavirus
K50	Crohn’s disease
K51	Ulcerative colitis
Biologic/Small Molecule: RxNorm Codes	
709271	Certolizumab:
847083	Ustekinumab:
191831	Infliximab:
327361	Adalimumab:
1538097	Vedolizumab:
1357536	Tofacitinib:
2288236	Ozanimod:
1431641	Golimumab:
Immunomodulators: RxNorm Codes	
6851	Methotrexate:
1256	Azathioprine:
Systemic Steroids: RxNorm Codes	
8640	Prednisone
19381	Budesonide:
